# VISIT STROKE: non-inferiority of telemedicine-based neurological consultation for post-acute stroke patients – protocol of a prospective observational controlled multi-center study

**DOI:** 10.1186/s12913-024-11651-3

**Published:** 2024-10-17

**Authors:** Juliane Herm, Hebun Erdur, Annette Aigner, Johannes Hengelbrock, Anselm Angermaier, Agnes Flöel, Annegret Hille, Claudia Gorski, Stephan Kinze, Ingo Schmehl, Gordian J. Hubert, Hanni Wiestler, Timo Siepmann, Martin Arndt, Christoph Gumbinger, Miriam Heyse, Joachim E. Weber, Heinrich J. Audebert, for the ANNOTeM-network

**Affiliations:** 1 Klinik und Hochschulambulanz für Neurologie, Charité – Universitätsmedizin Berlin, Hindenburgdamm 30, 12203 Berlin, Germany; 2 Center for Stroke Research Berlin (CSB), Charité – Universitätsmedizin Berlin, Berlin, Germany; 3 Klinik für Neurologie, Asklepios Fachklinikum Teupitz, Teupitz, Germany; 4 Institute of Biometry and Clinical Epidemiology, Charité – Universitätsmedizin Berlin, Berlin, Germany; 5 Department of Neurology, University Medicine Greifswald, Greifswald, Germany; 6 German Center for Neurodegenerative Diseases, partner site, Rostock, Greifswald, Germany; 7 Unfallkrankenhaus Berlin, Berlin, Germany; 8 TEMPiS telestroke center, Department of Neurology, München Klinik gGmbH, Munich, Germany; 9 Dresden Neurovascular Center, Department of Neurology, Medical Faculty and University Hospital Carl Gustav Carus, Technische Universität Dresden, Dresden, Germany; 10 Department of Neurology, University Hospital Heidelberg, Heidelberg, Germany; 11 Clinical Research Unit, Berlin Institute of Health, Berlin, Germany; 12 Department of Neurology, Charité - Universitätsmedizin Berlin, Hindenburgdamm 30, 12203 Berlin, Germany

**Keywords:** Stroke, Telemedicine, Tele-stroke, Tele-visitation, Stroke unit, Post-acute stroke management

## Abstract

**Background:**

Telemedicine provides specialized medical expertise in underserved areas where neurological expertise is frequently not available on a daily basis for hospitalized stroke patients. While tele-consultations are well established in acute stroke assessment, the value of telemedicine-based ward-rounds in the subsequent in-patient stroke management is unknown.

**Methods:**

Four telemedicine stroke networks in Germany, implemented in eight out of 16 federal states, participate in this prospective observational multi-center study. We plan to enroll 523 patients hospitalized due to acute (suspected or confirmed) stroke or transient ischemic attack. Each recruited patient will receive both a tele-consultation and an on-site consultation at the same day within the first three days after hospital admission. We will test non-inferiority of telemedicine-based assessments in ward-rounds in terms of quality of medical assessment and recommendations for hospitalized stroke patients. The correctness of the medical assessment and recommendation is defined as positive evaluation (binary, correct vs. in-correct) of six out of six predefined quality indicators by at least two out of three blinded independent raters. The non-inferiority margin for the difference in proportions of correct assessments is set to 5%-points.

**Discussion:**

If non-inferiority of telemedicine-based ward-rounds compared to on-site ward-rounds by a neurologist were demonstrated, telemedicine-based neurological consultation for post-acute stroke patients may contribute to deliver evidence-based high-quality stroke care more easily in underserved regions.

**Trial registration:**

DRKS - DRKS00028671 (https://drks.de/search/de/trial/DRKS00028671; registration date 09-27-2022).

## Background

Telemedicine provides specialized medical expertise on a broad scale, and telemedicine stroke networks, along with cardiologic applications and tele-radiology, have been at the forefront of telemedicine implementation [1, 2]. Since the establishment of the first German telestroke network in 2001, telemedicine-assisted stroke treatment has become a systemically relevant component of health care in Germany, with approximately 40,000 tele-consultations (10% of acute stroke patients) conducted annually in over 200 interconnected hospitals [3].

Currently, the majority of stroke tele-consultations take place during the acute phase in the emergency department or in the pre-hospital phase [4, 5]. Telemedicine-supported acute stroke management (e.g. intravenous thrombolysis and referral for thrombectomy) has repeatedly shown to be associated with better quality of care and decision-making as well as improved clinical outcomes such as reduced mortality and disability [2, 6–10]. Yet, during the subsequent in-patient treatment in the post-acute phase, stroke patients are vulnerable to complications. Further diagnostic measures (e.g. ECG-monitoring, arterial vessel imaging, echocardiography) are part of stroke work-up. Based on the results, the treating physician initiates secondary prevention measures. Due to a shortage of neurologists particularly in rural hospitals, there is currently a lack of every-day available neurological expertise for hospitalized stroke patients especially on weekends. Potential consequences include insufficient identification of short-term risks or complications, incomplete assessment of stroke etiology as well as suboptimal secondary stroke prevention. These deviations from standard stroke care can increase risk of morbidity and mortality in stroke survivors because they compromise the beneficial effects of increasingly personalized stroke treatment [11].

If this approach proves to be feasible, it offers the chance not only to support uncovered regions but also to provide highly specialized expertise by a vascular neurologist.

## Methods

### Study objectives

The primary objective is to assess non-inferiority of telemedicine-based ward-rounds by a stroke neurologist of a telestroke center compared to on-site ward-rounds conducted by a neurologist in terms of correctness of medical assessments and recommendations for hospitalized patients with suspected stroke or transient ischemic attack (TIA). In the event of demonstrating non-inferiority, we will assess superiority of telemedicine-based consultation compared to on-site ward-rounds regarding the same primary endpoint. In additional secondary analyses, we will compare the correctness of medical assessments and recommendations in each of the predefined quality indicators for non-inferiority and, if non-inferiority is established, for superiority. Furthermore, we will evaluate the overall quality of both assessments and recommendations.

### Study population

We will conduct this multicenter study in four telemedicine stroke networks (ANNOTeM, FAST, SOS-TeleNET, TEMPiS) in Germany, implemented in eight out of sixteen federal states (Baden-Württemberg, Bavaria, Brandenburg, Hesse, Mecklenburg-Western Pomerania, Rhineland-Palatinate, Saxony-Anhalt, Saxony).

### Inclusion and exclusion criteria

Patients over 18 years of age, hospitalized due to an acute stroke, including confirmed or suspected ischemic or hemorrhagic stroke, and TIA, are eligible for inclusion. An initial neurological consultation as part of the admission either in person or via telemedicine must have taken place before study inclusion. Patients will only be included given their written informed consent to study participation, or consent of their legal representative. Time from symptom onset to hospital admission > 72 h is an exclusion criterion.

### Study design

All recruited patients will receive both an on-site ward-round by a neurologist and a tele-consultation by a neurologist. The ward-round and tele-consultation will take place independently of each other, within three days after hospital admission. Both consultations are conducted on the same workday, usually in close temporal succession (less than 6 h apart). This will ensure that a highly similar patient status is assessed and available for comparative evaluation in the same patient. The neurologist conducting the on-site ward-rounds and the tele-neurologist will perform their ward-rounds independently and will not have access to the documentation of the counterpart consultation. The study nurse will enter the pseudonymized data of ward-round documentation along with findings from the patient’s medical records into a web-based, data protection-compliant study database (RedCap [12]). The tele-neurologist will not transmit the recommendation of the tele-consultation to the treating physicians on-site in order to avoid information bias, except there is an acute medical condition requiring immediate action. Three independent neurologists, blinded to the consultation mode, will evaluate the correctness of the ward-round documentation and recommendations based on predefined quality indicators. Blinding will be achieved by transcribing each recommendation into a uniform template, changing or deleting wordings that may unblind the reviewer as appropriate (e.g. “via camera”, “according to network guidelines”).

### Outcome definitions

#### Primary outcome

The primary outcome is the overall correctness of the medical assessment and recommendation regarding six quality indicators (binary outcome, correct vs. incorrect). A blinded adjudication committee will evaluate the documented medical assessment and recommendations for each quality indicator. Each of the three independent raters will rate both the on-site and the telemedicine-based ward-round reports of each case. For rating purposes, the reports will be transcribed in a way that adjudicators cannot identify the way of neurological assessment (on-site or telemedicine-based). We define correctness of medical assessment and recommendation as the positive evaluation of six out of six predefined quality indicators by at least two out of three blinded independent raters. The blinded adjudication committee will consist of six to eight board-certified neurologists with vascular expertise who will be independent by not being employed by or affiliated to any of the network hospitals. They are based in Berlin (Germany), Neuss (Germany), Oldenburg (Germany), Potsdam (Germany), Otago (New Zealand) and Aarhus (Denmark).

The six predefined quality indicators are the following:

1. Etiological classification: Classification regarding the diagnosis (ischemic vs. hemorrhagic stroke, TIA, stroke mimic) as well as (presumed) stroke etiology (for ischemic stroke based on TOAST criteria; for hemorrhagic stroke, anatomical classification of hemorrhage). Both classifications will have to be correct in order to be rated as correct.
2. Plausibility and consistency of documented neurological examination and documented NIHSS (National Institutes of Health Stroke Scale): Plausibility and consistency will be judged based on the documented initial neurological assessment in the emergency department as well as the discharge documentation.
3. Risk assessment: Detection of patients at risk of early stroke recurrence and development of complications: e.g. patients with stenosis of the brain-supplying arteries, with fluctuating neurological deficits / early neurological deterioration, with swallowing disorders and risk of developing aspiration pneumonia, patients at risk of falls, patients at risk of delirium.
4. Recommended diagnostic measures: Recommendation of further diagnostic measures needed for correct determination of stroke etiology and specific treatment recommendations.
5. Choice of secondary prevention: Recommendation based on current clinical findings and individual risk profile.
6. Recommended rehabilitation / follow-up care / prognosis: Recommendation of rehabilitation / follow-up care based on neurological deficits in activities of daily living and neurological prognosis.

#### Secondary outcomes

Secondary outcomes will be the correctness of medical assessment and recommendations in each of the six quality indicators individually. Again, we define correctness of recommendation as the positive evaluation of the respective quality indicator by at least two out of three raters. Further secondary outcomes will include comparisons of professional quality of the medical assessment and recommendations on a visual analogue scale ranging from 1 (assessment/ recommendation of physician A is strongly preferable) to 11 (assessment/ recommendation of physician B is strongly preferable), where 6 indicates that both are equal) in terms of thoroughness and correctness.

### Sample size calculation

As the primary analysis involves the comparison of two correlated proportions, we used the method proposed by Nam for sample size calculation [13]. We assumed the proportion of correct assessments by the on-site neurologist and tele-neurologist to be 80% each. The proportion of assessments where the on-site neurologist and tele-neurologist agree (including both correct and incorrect assessments) is assumed to be 80%. The analysis is then the difference between the two proportions, assuming a non-inferiority margin of 5%. With a power of 80% and a one-sided significance level of 5%, a sample size of 507 participants is needed. Assuming a proportion of missing values of 3% (e.g., when the on-site neurologist cannot examine the patient), a target sample size of 523 is aimed for. The sample size calculation was performed using PASS 16.0.7.

### Statistical analysis

We will analyze the primary outcome with a non-inferiority test with a significance level of 5% for the difference between two correlated proportions, based on the method proposed by Nam [13]. For the primary and all secondary endpoints, we will calculate two-sided 90% confidence intervals (CI) for this risk difference with a method based on the same score function as the Nam test [14]. Therefore, the comparison of the lower bound of the CI to the non-inferiority margin leads to the same test decision as the one-sided hypothesis test at the 5% significance level [15]. If non-inferiority of the proportion of correct recommendations by the tele-neurologist can be demonstrated, the secondary analysis will examine the superiority of tele-medicine-based ward-rounds based on the same CI.

A sensitivity analysis will be based on generalized estimating equations (GEE) [14], a regression-type analysis which accounts for the clustering of observations within patients. Such models can incorporate patients for whom only one assessment took place, and additionally they can be used to take into account potential center-effects and the sequence of assessments.

## Discussion

If the non-inferiority or even superiority of telemedicine-based consultations compared to on-site ward-rounds by a neurologist were demonstrated, providing high-quality stroke care would be facilitated in underserved regions. Moreover, the concept of telemedicine-based ward-rounds may possibly be transferrable to other medical specialties, thus providing new perspectives for healthcare delivery in rural areas.

Our study investigates the quality of care on multiple levels such as determination of stroke diagnosis and etiology, identification and treatment of post-stroke complications and implementation of secondary prevention measures adapted to the specific etiology of stroke. In case of showing non-inferiority of the telemedicine approach, evidence-based specialized vascular expertise in diagnosis and treatment of stroke could be made available to a larger number of patients, which may result in a reduction of morbidity and mortality after stroke. Furthermore, specialized vascular expertise would benefit patients especially in rural areas, thereby partially compensating for the shortage of healthcare professionals in these regions [16]. By transferring knowledge from major stroke centers, changes in guidelines and recent study findings could be implemented more quickly in a greater number of hospitals and regions.

### Strengths and limitations

The main strength of this study is its clinical practice approach. The results can be directly transferred to clinical routine in Germany and probably to other health care systems. Due to the high geographic coverage of regions in eight out of 16 federal states of Germany, the results of this multicenter study are likely generalizable to other regions and populations supporting the external validity of this study. Second, at least six raters will assess study outcomes independently, strengthening the internal validity of observations. Furthermore, the blinding of tele-neurologists and on-site neurologists to each other’s recommendation as well as blinding of raters to the consultation mode will reduce risk of bias. Finally, the planned sensitivity analyses will allow the inclusion of patients with only one consultation and furthermore account for potential center-effects and sequence of assessments.

We acknowledge several limitations: First, assessments and recommendations by different physicians may differ and deciding on correctness may be difficult in some cases. Therefore, we plan on performing three ratings per ward-round report, and we will assign raters randomly in order to avoid bias. Second, patients or their legal representatives have to provide written informed consent prior to inclusion, thus introducing a potential selection bias to the study – usually increasing the proportion of mildly affected patients. Third, this study is powered on non-inferiority of assessment and recommendations. Therefore, no conclusions can be drawn on patient outcomes such as functional outcome, disability, and deaths. Fourth, blinding of the raters may prove difficult, when the style or content of recommendation vary greatly between tele-neurologists and on-site neurologists.

## Data Availability

No datasets were generated or analysed during the current study.

## Abbreviations

CI: Confidence intervals
GEE: Generalized estimating equations
NIHSS: National Institutes of Health Stroke Scale
TIA: Transient ischemic attack
